# Pharmacokinetics of anastrozole and tamoxifen alone, and in combination, during adjuvant endocrine therapy for early breast cancer in postmenopausal women: a sub-protocol of the ‘Arimidex™ and Tamoxifen Alone or in Combination’ (ATAC) trial

**DOI:** 10.1054/bjoc.2001.1925

**Published:** 2001-08

**Authors:** The ATAC Trialists' Group

**Affiliations:** CRC and UCL Cancer Trials Centre, University College London, Stephenson House, 158-160 N Gower Street, London, NW1 2ND, UK

**Keywords:** arimidex, tamoxifen, aromatase inhibitor, oestrogen, oestradiol, breast cancer

## Abstract

The ATAC trial evaluates in a randomized, double-blind design, Arimidex™ (anastrozole) alone or in combination with tamoxifen, relative to tamoxifen alone as 5-year adjuvant treatment in postmenopausal women with early breast cancer. Patients included in the pharmacokinetic (PK) sub-protocol had been in ATAC for ≥3 months, taking their medication in the morning and were 100% compliant for the preceding 14 days. Blood samples were collected 24 ± 4 h after last dose. Trough (C_min_) plasma concentrations of anastrozole, tamoxifen and desmethyltamoxifen (DMT) were measured by validated methods. The PK results were based on a total of 347 patients (131 anastrozole (1 mg o.d.), 111 tamoxifen (20 mg o.d.), 105 anastrozole and tamoxifen (1 and 20 mg o.d. respectively)). The geometric mean steady-state trough plasma concentrations of tamoxifen and DMT were statistically equivalent in patients receiving tamoxifen alone or in combination with anastrozole: geometric mean tamoxifen = 94.8 ng ml^−1^and 95.3 ng ml^−1^in tamoxifen alone and combination groups, respectively; geometric mean DMT = 265.1 and 277.6 ng ml^−1^in the tamoxifen and anastrozole and tamoxifen groups, respectively. The geometric mean anastrozole levels were 27% lower (90% Cl 20–33%;*P*< 0.001) in the presence of tamoxifen than with anastrozole alone. Baseline plasma oestradiol levels were not obtained in the PK sub-protocol, however, such information was available from a similar ATAC sub-protocol, which evaluated bone mineral density. Mean oestradiol levels were 21.3, 19.3, and 21.6 pmol l^−1^prior to treatment and 3.7, 20.9 and 3.6 pmol l^−1^after 3 months in the anastrozole, tamoxifen, and combination groups, respectively (*n* = 167). On-treatment values were below the detection limit (3 pmol l^−1^) in 43.6 and 38.5% of the anastrozole alone and anastrozole in combination with tamoxifen groups, respectively. As a result of (a) the lack of effect of anastrozole on tamoxifen and DMT levels and (b) the observed fall in blood anastrozole levels having no significant effect on oestradiol suppression by anastrozole, we conclude that the observed reduction in anastrozole levels by tamoxifen is unlikely to be of clinical significance when anastrozole and tamoxifen are administered together. © 2001 Cancer Research Campaign http://www.bjcancer.com


British Journal of Cancer (2001) 85(3), 317–324
© 2001 Cancer Research Campaign
doi: 10.1054/ bjoc.2001.1925, available online at http://www.idealibrary.com on IDEAL®

http://www.bjcancer.com

### Pharmacokinetics of anastrozole and tamoxifen alone, and in combination, during adjuvant endocrine therapy for early breast cancer in postmenopausal women: a sub-protocol of the 'Arimidex™ and Tamoxifen Alone or in Combination' (ATAC) trial

**The ATAC Trialists' Group**[1]

CRC and UCL Cancer Trials Centre, University College London, Stephenson House, 158-160 N Gower Street, London NW1 2ND, UK

**Summary** The ATAC trial evaluates in a randomized, double-blind design, Arimidex™ (anastrozole) alone or in combination with tamoxifen, relative to tamoxifen alone as 5-year adjuvant treatment in postmenopausal women with early breast cancer. Patients included in the pharmacokinetic (PK) sub-protocol had been in ATAC for ≥ 3 months, taking their medication in the morning and were 100% compliant for the preceding 14 days. Blood samples were collected 24 ± 4 h after last dose. Trough ($C_{min}$) plasma concentrations of anastrozole, tamoxifen and desmethyltamoxifen (DMT) were measured by validated methods. The PK results were based on a total of 347 patients (131 anastrozole (1 mg o.d.), 111 tamoxifen (20 mg o.d.), 105 anastrozole and tamoxifen (1 and 20 mg o.d. respectively)). The geometric mean steady-state trough plasma concentrations of tamoxifen and DMT were statistically equivalent in patients receiving tamoxifen alone or in combination with anastrozole: geometric mean tamoxifen = 94.8 ng $ml^{-1}$ and 95.3 ng $ml^{-1}$ in tamoxifen alone and combination groups, respectively; geometric mean DMT = 265.1 and 277.6 ng $ml^{-1}$ in the tamoxifen and anastrozole and tamoxifen groups, respectively. The geometric mean anastrozole levels were 27% lower (90% CI 20–33%; $P < 0.001$) in the presence of tamoxifen than with anastrozole alone. Baseline plasma oestradiol levels were not obtained in the PK sub-protocol, however, such information was available from a similar ATAC sub-protocol, which evaluated bone mineral density. Mean oestradiol levels were 21.3, 19.3, and 21.6 pmol $l^{-1}$ prior to treatment and 3.7, 20.9 and 3.6 pmol $l^{-1}$ after 3 months in the anastrozole, tamoxifen, and combination groups, respectively ($n$ = 167). On-treatment values were below the detection limit (3 pmol $l^{-1}$) in 43.6 and 38.5% of the anastrozole alone and anastrozole in combination with tamoxifen groups, respectively. As a result of (a) the lack of effect of anastrozole on tamoxifen and DMT levels and (b) the observed fall in blood anastrozole levels having no significant effect on oestradiol suppression by anastrozole, we conclude that the observed reduction in anastrozole levels by tamoxifen is unlikely to be of clinical significance when anastrozole and tamoxifen are administered together. © 2001 Cancer Research Campaign http://www.bjcancer.com

**Keywords**: arimidex; tamoxifen; aromatase inhibitor; oestrogen; oestradiol; breast cancer

The link between oestrogen and the growth and development of some breast cancers has long been recognized (Beatson, 1896; Boyd, 1900), and there is substantial evidence that circulating oestrogens promote the proliferation of breast cancer (Jordan, 1976; Knazek et al, 1977). Many current therapies for breast cancer involve hormonal manipulation, with oestrogen-deprivation of the tumour being an established method of treatment. Drug therapy can achieve this goal either by antagonizing the effects of circulating oestrogens on receptors at the tumour site using anti-oestrogens, or by reducing the level of oestrogen within the body using drugs such as aromatase inhibitors (Howell and Dowsett, 1997).

Tamoxifen is currently the endocrine drug of choice for the treatment of early breast cancer in patients with oestrogen receptor-positive disease. The effects of tamoxifen in breast cancer appear to be primarily related to its anti-oestrogenic activity/oestrogen-receptor (ER) antagonist activity, although other mechanisms of action may also be important (Jaiyesimi et al, 1995).

Aromatase inhibitors are a class of compounds that act systemically to inhibit oestrogen synthesis in tissues, by inhibiting the enzyme aromatase, which catalyses the conversion of androgens (androstenedione and testosterone) to oestrogens (oestrone and oestradiol). In postmenopausal women, oestrogens are mostly derived from adrenal androgens being converted by aromatase in peripheral tissues (Grodin et al, 1973; Brodie and Njar, 1998). Aromatase is also present in many breast carcinomas where it may provide an important source of oestrogenic stimulation (Miller and O'Neill, 1987).

Anastrozole (Arimidex) is a third-generation non-steroidal aromatase inhibitor, which is both potent and selective for the enzyme. It is given orally, is well tolerated, and provides almost complete oestrogen suppression both systemically and intratumourally (Geisler et al, 1996, 1999). Anastrozole is effective in treating advanced breast cancer (Buzdar et al, 1996) and at a dose of 1 mg once-daily, it significantly increases survival time

*Received 20 September 2000*
*Revised 22 April 2001*
*Accepted 2 May 2001*

*Correspondence to:* The ATAC Secretariat

[1]Contributors to the trial are listed in Appendices I, II, and III in the Acknowledgements section.

compared with megestrol acetate (median follow-up 31 months) (Buzdar et al, 1998). Recently, it was shown to be at least as effective as tamoxifen (20 mg o.d.) for first-line treatment of postmenopausal advanced breast cancer (Bonneterre et al, 2000; Nabholtz et al, 2000). One of these studies was conducted in North America and the other in Europe, Australia, New Zealand, South Africa and South America. In patients with tumours known to be ER-positive and/or progesterone receptor (PgR)-positive, anastrozole significantly prolonged time to disease progression compared with tamoxifen (median values of 10.7 and 6.4 months for anastrozole and tamoxifen, respectively, $P = 0.022$) (Nabholtz et al, 2000).

From the different and potentially complementary modes of action of tamoxifen and anastrozole, it is possible that using both agents in combination may provide a more effective method of treating breast cancer in postmenopausal women. A study referred to as the 'Arimidex and Tamoxifen Alone, or in Combination (ATAC)' trial is currently underway to investigate this hypothesis. The trial, which has recruited over 9300 patients, is designed to compare the efficacy and safety of anastrozole and the combination of anastrozole plus tamoxifen with that of tamoxifen alone, as adjuvant treatment for postmenopausal women with early breast cancer, who have completed their primary therapy (surgery with or without radiotherapy and/or chemotherapy) (Houghton and Baum, 1998).

Before initiating this large clinical trial programme it was noted that the prototype aromatase inhibitor, aminoglutethimide, reduced blood levels of tamoxifen by more than 50% when given in combination (Lien et al, 1990). Hence a pilot study was conducted to determine whether or not anastrozole had any effect on tamoxifen blood levels, and thus, potentially any impact on the clinical efficacy of tamoxifen (Dowsett et al, 1999a). That study showed that anastrozole did not affect the pharmacokinetics of tamoxifen when the 2 drugs were given in combination to postmenopausal women with early breast cancer. Additionally, anastrozole remained an effective suppressant of oestradiol when given in combination with tamoxifen (Dowsett et al, 1999a). However, no assessment was made of the major active metabolite of tamoxifen, desmethyltamoxifen, which has a similar pharmacological profile to tamoxifen but with concentrations approximately 50% higher than the parent drug. In addition, the effect of tamoxifen on the pharmacokinetics of anastrozole could not be directly assessed with the design of that study. However, preclinical data in rats suggest that there is no synergism between anastrozole and tamoxifen (Brodie et al, 1999, the half-life of anastrozole in the rat is very different compared with a patient (8 hours in the rat and 50 hours in a patient); Dukes, 1997). Therefore, it cannot be assumed that the lack of synergistic activity of these agents in a rat can be extrapolated to the clinical situation.

We now report data from an assessment of the pharmacokinetics of both anastrozole and tamoxifen when given alone or in combination as adjuvant endocrine therapy for early breast cancer as determined in a sub-protocol of the ATAC trial. Following the initial pharmacokinetic assessments, further data were taken from a separate ATAC protocol in order to evaluate oestradiol suppression.

### PATIENTS AND METHODS

### Study design

This was a sub-protocol of the ATAC trial, which is a randomized, double-blind, multicentre study. The sub-protocol was designed to compare the pharmacokinetics of the combination of anastrozole plus tamoxifen with those of anastrozole alone or tamoxifen alone when used as adjuvant treatment for early breast cancer in postmenopausal women (Houghton and Baum, 1998).

The study was designed and monitored to comply with the ethical principles of Good Clinical Practice (GCP) in accordance with the Declaration of Helsinki. All patients included in the study had given their informed consent.

The primary assessments were the steady-state trough plasma concentrations of tamoxifen, desmethyltamoxifen, and anastrozole, measured at 24 ± 4 hours after the previous dose of treatment.

There was no intent to measure baseline or on-treatment oestradiol levels in the PK sub-protocol, but measuring the effects of anastrozole with or without tamoxifen on oestradiol levels became desirable after the pharmacokinetic data became available. Thus, this information was sought from a separate ATAC sub-protocol, which looked at bone mineral density in a total of 167 patients. This cohort was chosen since this sub-protocol required blood samples to be taken from patients at baseline and 3 months following treatment, allowing the impact of drugs on the proportional suppression of oestradiol to be established and compared between the treatments.

### Patient population

All patients were initially recruited into the ATAC trial and were required to be postmenopausal, have a diagnosis of early breast cancer, have completed all primary treatment and chemotherapy (if given), and be suitable to receive adjuvant endocrine therapy.

The inclusion criteria for the patients recruited to both the bone mineral density and pharmacokinetic studies were the same. To be eligible for this pharmacokinetics subprotocol, all patients had to have been participating in the ATAC trial for at least 3 months, have taken their medication in the morning and have been fully compliant over the preceding 14 days before the study assessments were performed. Each trial investigator or pharmacist was responsible for the maintenance of drug accountability records, which consisted of documentation of receipt, dispensing, and the return of trial medication. Drug dispensing was documented by attaching the tear-off portion of the drug label to each patient record (or case report form in North America) and this was initialled and dated by the investigator. At the end of the trial, patients were asked to return all unused drugs and a note of the number of returned tablets was entered on to the patient record or case report form. The completed forms were then signed off and dated by the investigator.

Exclusion criteria for the pharmacokinetic sub-protocol were concurrent treatment with diazepam, oral administration of ketoconazole or related antifungal compounds, or drugs that might affect steroid hormone status and/or tamoxifen steady-state levels (cytotoxic chemotherapy or other hormonal treatments for breast cancer). Exclusion criteria for the bone mineral density protocol above that of the main sub-protocol were patients who have: received hormone replacement therapy or bisphosphate therapy within the previous 12 months prior to recruitment; had a bone fracture in the previous 6 months prior to recruitment; chronic renal/liver impairments; malabsorption syndrome; endocrine disorders including hyperthyroidism, untreated thyroid disease, Cushing's syndrome or pituitary disease; or patients taking corticosteroids or anti-convulsants. Demographic data from this and other sub-protocols, as well as from the ATAC trial as a whole, will be published in the future.

 © 2001 Cancer Research Campaign

### Treatment programme

Patients enrolled in the ATAC trial were randomized on a 1:1:1 basis into 1 of 3 treatment schedules (active anastrozole 1 mg o.d. and tamoxifen placebo o.d., active tamoxifen 20 mg o.d. and anastrozole placebo o.d., or active anastrozole 1 mg o.d. and active tamoxifen 20 mg o.d.). Patients at 24 centres from 6 countries that agreed to participate in the sub-protocol were screened for additional eligibility criteria for entry 3 months after their randomization into the ATAC trial. In order that the blinding of the ATAC trial would not be compromised, patients were issued with a unique identifying number specific to this sub-protocol, which could not be linked to the patient identifier in the ATAC trial.

### Assessments

In the pharmacokinetic sub-protocol, patients were required to give only one 15 ml blood sample, after steady-state levels had been achieved (3 months after treatment randomization). The elimination half-lives of tamoxifen, desmethyltamoxifen, and anastrozole are 4–7 days (Adam, 1981a, 1981b); 6.7–12 days (Adam, 1981a, 1981b; Mould et al, 1986a, 1986b; De-vos et al, 1989), and 40–50 hours (Yates et al, 1996), respectively. Therefore, by taking the samples 3 months after treatment randomization ensured that steady-state would be reached by this time. In this sample, trough ($C_{min}$) plasma concentrations of anastrozole, tamoxifen, and desmethyltamoxifen were measured. For the purposes of this study, trough levels were defined as the plasma concentration of drug 24 hours after the previous dose. However, for practical reasons blood samples withdrawn between 20 and 28 hours (24 ± 4 hours) after the last dose were acceptable. Because trough levels were required, it was important that no medication was taken on the day of assessment until after the blood sample was withdrawn. If a patient had taken medication within the previous 24 ± 4 hours, she was asked to give her blood sample at another visit.

Plasma levels of tamoxifen and desmethyltamoxifen were analysed according to the high-performance liquid chromatography (HPLC) method described by Johnston et al (1993). Overall, recovery varied from 79% to 93% and was corrected for by the internal recovery control. The intra-assay coefficient of variation was 2.2% at a mean level of 135 ng $ml^{-1}$. The sensitivity limit for the detection of tamoxifen was 0.5 ng $ml^{-1}$ and for desmethyltamoxifen was 0.275 ng $ml^{-1}$. Plasma levels of anastrozole were determined using a validated method involving solvent extraction, capillary gas chromatography, and separation with electron capture detection (ECD) (Bock et al, 1997). The sensitivity limit for the detection of anastrozole was 3.0 ng $ml^{-1}$.

Oestradiol levels were assessed from a blood sample taken at baseline and at 3 months following treatment; the analytical method for this had a sensitivity limit of 3 pmol $l^{-1}$ and has been previously described (Dowsett et al, 1987).

### Statistical analysis

The primary assessments of the sub-protocol were the steady-state plasma concentrations of anastrozole (Bock et al, 1997), tamoxifen and desmethyltamoxifen. For the steady-state $C_{min}$ plasma levels of tamoxifen and desmethyltamoxifen, comparisons were made between samples from patients treated with either tamoxifen alone or with anastrozole plus tamoxifen. For the steady-state $C_{min}$ plasma levels of anastrozole, comparisons were made between samples from patients treated with either anastrozole alone or anastrozole in combination with tamoxifen.

To demonstrate equivalence between 2 treatment groups in terms of plasma levels, the 90% confidence interval for the ratio of the geometric means (combination/monotherapy) was required to lie within the interval > 0.8 to 1.25. A population of 110 patients per treatment arm was required to demonstrate equivalence of the plasma levels of tamoxifen, desmethyltamoxifen, and anastrozole with a minimum 90% power.

All comparisons were analysed using analysis of variance. All plasma levels were log (base e) transformed prior to analysis. The results were back transformed and presented in terms of the geometric means. The treatment effect was the ratio of the geometric mean of the combination group divided by the geometric mean of the associated monotherapy group. The appropriate equivalence test criteria was the 90% confidence interval for the ratio of geometric mean values. Equivalence was concluded if the 90% confidence interval lay entirely within the (0.8, 1.25) interval.

For oestradiol levels, the comparison between the anastrozole and combination treatment groups was assessed using analysis of variance. The percentage reduction in oestradiol levels was determined with the baseline fitted as a covariate. Oestradiol levels were log (base e) transformed prior to analysis. The results were back transformed and presented in terms of the adjusted (for baseline oestradiol) geometric least square means (GLS Means), representing the oestradiol level at 3 months as a proportion of the level at baseline. The 90% confidence interval of the ratio of GLS Means was calculated along with estimated percentage reductions in oestradiol in each treatment group.

### RESULTS

### Patient characteristics

A total of 357 patients (138 anastrozole 1 mg o.d., 113 tamoxifen 1 mg o.d., and 106 anastrozole co-administered with tamoxifen) were recruited into this sub-protocol from 24 international centres. The groups were well balanced with respect to demographic characteristics (Table 1). 10 patients were excluded from the pharmacokinetic analyses because the results of laboratory tests did not correspond with those that would be anticipated from their recorded treatment allocation, highlighting uncertainties in the actual treatment received. In 2 patients recorded as receiving anastrozole alone, tamoxifen was identified at a concentration in the range of those seen clinically; one patient recorded as receiving anastrozole alone had undetectable anastrozole levels; 5 patients recorded as receiving anastrozole alone also had trace levels of tamoxifen; and in 2 patients recorded as receiving tamoxifen alone there were detectable low levels of anastrozole. The demographic characteristics of the patients enrolled in the bone sub-protocol, whose blood samples were analysed for plasma oestradiol levels, were comparable to those of the patients in the pharmacokinetic sub-protocol of the ATAC trial. Because the study looking at bone mineral density is ongoing, we were unable to break the drug code and carry out statistical tests. However, the demographics of the bone density sub-protocol were comparable with the patients from the main ATAC trial.

© 2001 Cancer Research Campaign

**Table 1** Patient characteristics

| Demographic characteristics | Anastrozole (1 mg o.d.) | Tamoxifen (20 mg o.d.) | Anastrozole + tamoxifen | Bone sub protocol |
|---|---|---|---|---|
| Number of patients | 138 | 113 | 106 | 302 |
| Age (years) | | | | |
| *n* | 138 | 111 | 105 | 301 |
| Median | 65 | 63 | 62 | 65 |
| Minimum | 42 | 43 | 40 | 42 |
| Maximum | 87 | 88 | 84 | 86 |
| BMI | | | | |
| *n* | 130 | 107 | 105 | 288 |
| Median | 26.7 | 26.3 | 26.9 | 28 |
| Minimum | 22.7 | 23.5 | 20.3 | 17.6 |
| Maximum | 54.9 | 36.2 | 32.1 | 52.3 |
| Origin (number of patients) | | | | |
| Caucasian | 132 (95.7%) | 104 (92.0%) | 102 (96.2%) | 272 (90.1%) |
| Black/Afro-Caribbean | 2 ( 1.4%) | 1 (0.9%) | 1 (0.9%) | 4 (1.3%) |
| Hispanic | 1 ( 0.7%) | 1 (0.9%) | 0 | 2 (0.6%) |
| Asian | 1 ( 0.7%) | 0 | 0 | 0 |
| Mixed | 2 ( 1.4%) | 4 (3.5%) | 1 (0.9%) | 17 (6.2%) |
| Other | 0 | 0 | 1 (0.9%) | 2 (0.6%) |
| Not recorded | 0 | 3 (2.7%) | 1 (0.9%) | 5 (1.8%) |

### Plasma concentrations of tamoxifen and desmethyltamoxifen

There was very little difference between the mean steady-state trough plasma concentrations of tamoxifen and desmethyltamoxifen in patients receiving tamoxifen when compared with those of patients receiving anastrozole in combination with tamoxifen (Table 2). The mean level of tamoxifen in both groups was approximately 100 ng ml$^{-1}$ and levels of desmethyltamoxifen were almost 3-fold those of tamoxifen. Fewer values of desmethyltamoxifen were available since a batch of analyses failed quality control criteria and insufficient sample was available for repeat analysis. The ratio of the geometric mean values ((anastrozole and tamoxifen group)/tamoxifen group) was 1.01 for tamoxifen (90% confidence interval from 0.91 to 1.11), and 1.05 for desmethyltamoxifen (90% confidence interval from 0.94 to 1.16). This indicates that the tamoxifen and desmethyltamoxifen blood levels measured in patients receiving tamoxifen alone were equivalent to those in patients receiving tamoxifen in combination with anastrozole.

### Plasma concentrations of anastrozole

The mean steady-state trough plasma concentration of anastrozole in patients receiving anastrozole monotherapy was 37.4 ± 15.2 ng ml$^{-1}$. In patients co-administered with tamoxifen and anastrozole, the mean steady-state trough plasma concentration of anastrozole was found to be 27.7 ± 11.3 ng ml$^{-1}$ (Table 2).

**Table 2** Steady-state trough plasma concentrations ($C_{min}$) of tamoxifen, desmethyltamoxifen and anastrozole

| | Tamoxifen (20 mg o.d.) (*n* = 111) | Anastrozole (1 mg o.d.) and tamoxifen (20 mg o.d.) (*n* = 105) | Anastrozole (1 mg o.d.) (*n* = 131) |
|---|---|---|---|
| **Tamoxifen** | | | |
| *n* | 104 | 99 | – |
| Arithmetic mean ± SD (ng/ml) | 103.8 ± 40.9 | 103.8 ± 45.6 | – |
| Geometric mean | 94.8 | 95.3 | – |
| Ratio of geometric mean | | | |
| (anastrozole+tamoxifen)/tamoxifen | 1.01 | | |
| 90% CI (lower-upper) | (0.91–1.11) | | |
| **Desmethyltamoxifen** | | | |
| *n* | 76 | 76 | – |
| Arithmetic mean ± SD (ng/ml) | 286.6 ± 107.8 | 293.8 ± 98.9 | – |
| Geometric mean | 265.1 | 277.6 | – |
| Ratio of geometric mean | | | |
| (anastrozole+tamoxifen)/tamoxifen | 1.05 | | |
| 90% CI (lower-upper) | (0.94–1.16) | | |
| **Anastrozole** | | | |
| *n* | – | 104 | 130 |
| Arithmetic mean ± SD (ng/ml) | – | 27.7 ± 11.3 | 37.4 ± 15.2 |
| Geometric mean | – | 25.5 | 34.7 |
| Ratio of geometric mean | | | |
| (anastrozole+tamoxifen)/anastrozole | | 0.73 | |
| 90% CI (lower-upper) | | (0.67–0.80) | |

 *© 2001 Cancer Research Campaign*

The ratio of the geometric mean values ((anastrozole and tamoxifen group)/anastrozole group) for anastrozole was 0.73 (90% confidence interval from 0.67 to 0.80). This indicates that the mean anastrozole levels were a mean 27% lower ($P < 0.001$) in the presence of tamoxifen than with anastrozole alone, and therefore equivalence could not be concluded.

### Plasma concentrations of oestradiol

Of the 167 patients whose plasma oestradiol concentrations were measured before and after 3 months' treatment, 55 patients were on anastrozole, 62 were on tamoxifen and 50 were on anastrozole plus tamoxifen. Geometric mean levels were 21.3, 19.3 and 21.6 pmol $l^{-1}$, respectively before treatment and 3.7, 20.9 and 3.6 pmol $l^{-1}$, respectively after 3 months' treatment (Figure 1). The GLS Means for the ratio of 3-month values to baseline values were 0.174 for anastrozole alone and 0.169 for anastrozole plus tamoxifen, indicating that percentage suppression was 82.6 and 83.1%, respectively. The ratio of the GLS Means ((anastrozole + tamoxifen)/anastrozole) was 0.97 and the 90% confidence interval was 0.86 to 1.10. The percentage of oestradiol concentrations below the assay detection limit was 43.6 for anastrozole alone and 38.5 for the combination. Thus, the effect of anastrozole on oestradiol suppression was very similar whether administered alone or in combination with tamoxifen; none of the small differences in the data approached statistical significance.

The pretreatment oestradiol levels showed a highly significant ($P = 0.0001$) positive correlation with weight and body mass index but no relationship with height or smoking status. There was a positive correlation with age, and this approached statistical significance ($P = 0.06$).

### DISCUSSION

The aim of the ongoing ATAC study is to compare the efficacy and safety of anastrozole alone with those of tamoxifen alone or the combination of anastrozole with tamoxifen, as adjuvant treatment for postmenopausal women with early breast cancer, who have completed their primary therapy (surgery with or without radiotherapy and/or chemotherapy). As part of the larger ATAC programme, a number of sub-protocols have been undertaken, of which the pharmacokinetic sub-protocol is reported here.

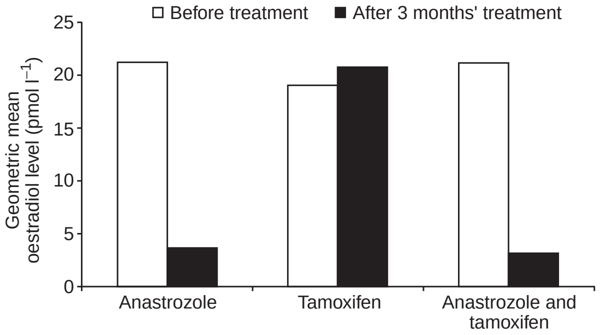

**Figure 1** Plasma concentrations of oestradiol measured before and after 3 months' treatment with anastrozole (1 mg o.d.), tamoxifen (20 mg o.d.), or anastrozole and tamoxifen in combination (1 mg o.d. and 20 mg o.d. respectively). Limit of detection for oestradiol, 3 pmol $l^{-1}$

Given the different modes of action of anastrozole and tamoxifen (Howell and Dowsett, 1997), there is a rationale for combining these 2 drugs due to the potential for synergy and increased efficacy. It was, therefore, necessary to determine whether or not the pharmacokinetics of the 2 drugs were affected when given in combination. It was considered particularly important to determine whether or not there was any effect on tamoxifen levels since tamoxifen is widely accepted to be the endocrine drug of choice for the treatment of early breast cancer in patients with oestrogen receptor-positive disease (Early Breast Cancer Trialists' Collaborative Group, 1992, 1998). The relevance of looking for possible interactions between anastrozole and tamoxifen was emphasized by the finding that the non-specific aromatase inhibitor, aminoglutethimide, had an interaction with tamoxifen resulting in a greater than 50% reduction in plasma tamoxifen levels (Lien et al, 1990). It was suggested that this reduction might have affected the clinical efficacy of tamoxifen when the 2 drugs were combined. This interaction was attributed to an action of aminoglutethimide whereby it induces the metabolism of tamoxifen. In contrast to the effects of aminoglutethimide on tamoxifen, however, anastrozole was previously found not to interact with the metabolism of tamoxifen in a study designed specifically to address that possibility (Dowsett et al, 1999a).

The findings of the present study were consistent with the results of this earlier pilot study (Dowsett et al, 1999a), showing that steady-state trough plasma tamoxifen concentrations were similar in patients treated with tamoxifen monotherapy or in combination with anastrozole. Mean tamoxifen levels were similar to those reported in that earlier study. Additionally, in the present study, the plasma levels of the biologically active metabolite, desmethyltamoxifen, were unaffected by the presence of anastrozole, indicating that no substantial effect on the major metabolic pathway of tamoxifen was induced by anastrozole.

This sub-protocol also set out to determine whether or not tamoxifen had any effect upon anastrozole blood levels, and, since the effectiveness of anastrozole as a treatment for breast cancer (Buzdar et al, 1998; Nabholtz et al, 2000) is related to its effectiveness as an oestrogen suppressant (Geisler et al, 1996), to compare the oestradiol suppression achieved by anastrozole alone with its effect in combination with tamoxifen. Co-administration of anastrozole with tamoxifen resulted in a mean 27% decrease in steady-state trough plasma anastrozole levels compared with those observed in patients administered anastrozole alone.

The finding of lower blood anastrozole levels when combined with tamoxifen administration is not unique to anastrozole; similar findings have been reported previously for the non-steroidal aromatase inhibitor, letrozole. In a study of 12 postmenopausal women with advanced breast cancer, the pharmacokinetics of combined treatment with tamoxifen (20 mg $day^{-1}$) and letrozole (2.5 mg $day^{-1}$) were examined. Plasma levels of letrozole were reduced by a mean 37.6% (90%Cl: 31.6–43.2%) during combination therapy ($P < 0.0001$) (Dowsett et al, 1999b). The mechanism was postulated to be a consequence of an induction of letrozole-metabolizing enzymes by tamoxifen, which would result in more rapid metabolism of letrozole and hence a reduction in plasma levels. However, this potential mechanism was not addressed in the study, nor has it been assessed in subsequent studies. It is possible that a similar mechanism may be responsible for the reduced plasma levels of anastrozole observed in this sub-protocol, although again this has not been investigated, and remains to be elucidated.

© 2001 Cancer Research Campaign

There are data available that suggest that the observed mean 27% reduction in anastrozole levels would not have significantly affected the degree of oestradiol suppression, and hence the clinical efficacy of anastrozole. In the pilot combination study (Dowsett et al, 1999a), suppression of serum oestradiol in patients who received anastrozole in combination with tamoxifen was to the limit of detection of the assay used (3 pmol $l^{-1}$). In an earlier study, administration of both 0.5 and 1 mg anastrozole once-daily achieved a greater than 80% reduction in the mean oestradiol levels (Yates et al, 1996). The present sub-protocol was designed to assess subjects who had already received treatment for 3 months prior to entry, such that pretreatment blood samples were not available to assess the relative suppression of oestrogen levels in the same patients in whom pharmacokinetic data were available.

Thus the opportunity was also taken to compare oestrogen levels in a further set of samples obtained from a separate sub-protocol of ATAC (to assess the effect of the treatment on bone metabolism) in which pretreatment and on-treatment samples at steady state were available. The demographic measures were comparable for both groups of patients. The inclusion criteria for the bone sub-protocol were the same as for this pharmacokinetic study, and although there were some exclusion criteria above those of the main ATAC sub-protocol, it is likely that the oestradiol-suppressing effects we report for the patients assessed for bone metabolism are the same as would be seen in the patients assessed for the pharmacokinetics of anastrozole and tamoxifen. The data revealed a very similar degree of suppression of oestradiol levels with anastrozole alone and in combination with tamoxifen, confirming expectations of no perceptible reduction in the pharmacological effectiveness of the lower anastrozole levels.

Tamoxifen alone had no significant effect on oestradiol levels. This is consistent with previous studies (Dowsett et al, 1999a), but was important to confirm in the present study since a pharmacological effect of tamoxifen on plasma oestradiol could have confounded the study of whether the lower plasma levels of anastrozole affected its effects on oestrogen suppression. The positive correlation of the pretreatment values of oestradiol with weight, BMI and (marginally) with age are also consistent with expectations since there are well-described positive relationships between peripheral aromatase activity and each of these parameters (Grodin et al, 1973; MacDonald et al, 1978). Demonstration of these relationships in this dataset suggests that the population studied may be considered representative of the postmenopausal population.

Overall the data from this study are reassuring in that the lack of effect of anastrozole on tamoxifen levels indicates that no reduction in the efficacy of tamoxifen would be anticipated in the combination arm of the ATAC trial. Thus the benefits of tamoxifen in this arm should not be lower than those in the tamoxifen monotherapy arm. The over-riding question, therefore, is whether or not combining anastrozole with tamoxifen will achieve increased efficacy when compared with tamoxifen alone. Definitive conclusions as to whether or not the efficacy of anastrozole is affected by the addition of tamoxifen cannot be made until the results of the main ATAC trial have been analysed. However, it does appear that the observed interaction between tamoxifen and anastrozole is unlikely to reduce the levels of anastrozole sufficiently to reduce its oestrogen-suppressive effects.

In conclusion, the results confirm that, in postmenopausal women with early breast cancer, co-administration of anastrozole does not affect steady-state trough plasma concentrations of tamoxifen. Steady-state trough plasma concentrations of the metabolite of tamoxifen, desmethyltamoxifen, are also unaffected. The observed reduction in the steady-state trough plasma concentrations of anastrozole in the presence of tamoxifen has no significant effect on the oestradiol suppressive effects of anastrozole. These results, therefore, indicate that the observed interaction is unlikely to be of clinical significance whenever anastrozole or tamoxifen are administered together. The results of the main ATAC trial will shed further light on these findings.

### ACKNOWLEDGEMENTS

Members of the Writing Group for this paper are asterisked.
Principal Investigator for the ATAC Pharmacokinetic Sub-protocol *Prof M Dowsett, The Royal Marsden Hospital, London, UK.

### APPENDIX 1 – ATAC TRIAL STEERING COMMITTEE MEMBERSHIP

Prof M Baum (Chairman and Principal Investigator for the main ATAC Trial), University College London, London, UK; Prof M Dowsett, The Royal Marsden Hospital, London, UK; Dr M Coibion, Institut Bordet, Bruxelles, Belgium; Prof AR Bianco, Universita Degli Studi Di Napoli Federico II, Napoli Italy; *Dr J Cuzick, Imperial Cancer Research Fund Labs, London, UK; Prof W D George, Western Infirmary, Glasgow, UK; Sr J Gray, Belfast City Hospital, Belfast, UK; *Dr A Howell, Christie Hospital and Holt Radium Institute, Manchester, UK; Mrs J Houghton, Dr N Williams, CRC and UCL Cancer Trials Centre, UCL Medical School, London, UK, UK; Prof J Sloane, Royal Liverpool University Hospital, Liverpool, UK; Dr J Tobias, The Meyerstein Institute of Clinical Oncology, Middlesex Hospital, London, UK; Dr A Buzdar, The University of Texas, M. D. Anderson Cancer Centre, Houston, USA; *Dr I Jackson, Dr T Sahmoud, Mr J Gallagher, Mr A Webster, AstraZeneca Pharmaceuticals, Macclesfield, UK.

### APPENDIX II – PRINCIPAL INVESTIGATORS IN THE ATAC PHARMACOKINETIC SUB-PROTOCOL

Prof. D Gangji, Erasme Hospital, Brussels, Belgium; Dr K Petrakova, Masarykuv onkologicky ústav, Brno, Czech Republic; Dr B Konopasek, Onkologicka klinika UK, Praha, Czech Republic; Dr P Mares, Onkologicka klinika UK, Praha, Czech Republic; Dr P Vodvarka, Radioterapeuticka klinika, Ostrava-Poruba, Czech Republic; Dr Ana Alcazar, Hospital Reynaldo dos Santos, Vila Franca de Xira, Portugal; Dr Ondina Campos, Maternidade Bissaya Barreto, Coimbra, Portugal; Dr A Maxwell, Durban, South Africa; Prof Goedhals, Nationale Hospital, Bloemfontein, South Africa; Dr D Hacking, Durban Oncoclogy Centre, Durban, South Africa; Dr G Landers, Parklands Hospital, Durban, South Africa; Dr L Smith, Nationale Hospital, Bloemfontein, South Africa; Dr DA Vorobiof, Sandton Oncology, Sandton, South Africa; Prof ID Werner, New Groote Schuur Hospital, Cape Town, South Africa; Professor R Blamey, City Hospital, Nottingham, UK; Prof R Coleman, Western Park Hospital, Sheffield, UK; Dr Robert J Grieve, Walsgrave Hospital, Coventry, UK; Dr T Hickish, Royal Bournemouth Hospital, Bournemouth, Dorset, UK; Prof A Howell, Christie Hospital and Withington Hospital, Manchester, UK; Mr JC Nicholls, St. Albans City

*© 2001 Cancer Research Campaign*

Hospital, St. Albans, Herts, UK; Mr S Nicholson, York District Hospital, York, UK; Mr S Raymond, St. Albans City Hospital, St Albans, Herts, UK; Mr A Salman, Worthing Hospital, Worthing, Sussex, UK; Dr J. Blum, Texas Oncology, PA, Dallas, TX, USA; Dr R. Clark, Hematology/Oncology Associates, Jackson, MI, USA; Dr A. Lyss, Missouri Baptist Cancer Center, St. Louis, MO, USA; Dr G. Miletello, Baton Rouge General Regional Cancer Center, Baton Roughe, LA, USA; Dr J. Sternberg, Clinical Investigation Specialists, Inc, Little Rock, AR, USA.

### APPENDIX III – ADDITIONAL TRIAL COMMITTEES AND COLLABORATIVE/OPERATIONAL GROUPS

### International Coordinating Committee

Prof J Forbes, Newcastle Mater Misericordiae Hospital, NSW, Australia; Dr M Coibion, Institut Bordet, Brussels, Belgium; Dr JM Nabholtz, Cross Cancer Institute, Edmonton, Alberta, Canada; Dr J P Guastalla, Centre Leon Berard, Lyon, France; Professor Dr W Distler, Universitatsklinikum Dresden, Dresden, Germany; *Professor Dr J G M Klijn, Dr. Daniel den Hoed Kliniek and University Hospital Rotterdam, Rotterdam, The Netherlands; Dr T Nagykalnai, Uzsoki U Hospital, Budapest, Hungary; Dr A Nicolucci, Givio Co-ordinating Centre, Consorzio Mario Negri Sud, Centro Di Ricerchi Farmacologichi, E Biomedichi, Chieta, Italy; Prof A R Bianco, Universita Federico II, Napoli, Italy; Dr M Constenla, Hospital Motelcelo, Pontevedra, Spain; Dr U Nylen, Radiumhemmet, Karolinska sjukhuset, Stockholm, Sweden; Prof A Howell, Christie Hospital, Manchester, UK; Mr R Sainsbury, Huddersfield Royal Infirmary, Huddersfield, UK; Prof R E Mansel, University of Wales College of Medicine, Cardiff, UK; Professor D George, Beatson Oncology Center, Western Infirmary, Glasgow, UK; Dr A U Buzdar, MD Anderson Center, University of Texas, Houston, TX, USA; Dr G Y Locker, Evanston Hospital, Kellogg Cancer Care Center, Evanston IL, USA.

### International Project Team

J Gallagher, I Jackson, T Sahmoud, AstraZeneca Pharmaceuticals, Macclesfield, UK; J Houghton, N Williams, CRC and UCL Cancer Trials Centre, UCL Medical School, London, UK; A Nicolucci, Mario Negri Institute, Chieta, Italy; S Pollard, Northern Yorkshire Clinical Trials Research Unit, Leeds, UK; P Stroner, SCTN Central Office, Information and Statistics Division, Edinburgh, UK.

### Independent Data Monitoring Committee

Dr M Buyse, International Institute for Drug Development (ID squared), Brussels, Belgium; Dr R Margolese, Mc Gill University, The Sir Mortimer B Davis Jewish General Hospital, Montreal, Quebec, Canada; Mr J M A Northover, ICRF Colorectal Cancer Unit, St Mark's Hospital, Harrow, Middlesex, UK.

### REFERENCES

Adam H (1981a) Pharmacokinetic studies with Nolvadex. *Rev Endocrine Related Cancer* Suppl 9: 131–143

Adam HK (1981b) What we know and don't know about the pharmacokinetics of tamoxifen. *Special Rep Chemotherap Effects of Nolvadex* 29–34 651.794 IC PL Symposium. Clinical applications of oncology San Diego 22 May 1981

Beatson GT (1896) On the treatment of inoperable cases of carcinoma in mamma; suggestion for new method of treatment with illustrative cases. *Lancet* **2**: 104–107

Bock MJH, Bara I, LeDonne N, Martz A and Dyroff M (1997) Validated assay for the quantification of anastrozole in human plasma by capillary gas chromatography/63Ni electron capture detection. *J Chrom B* **700**: 131–138

Bonneterre J, Thurlimann B, Robertson JFR, Krzakowski M, Mauriac L, Koralewski P, Vergote I, Webster A, Steinberg M and von Euler M, for the Arimidex Study Group (2000) Anastrozole versus tamoxifen as first-line therapy for advanced breast cancer in 668 postmenopausal women: results of the TARGET (Tamoxifen or Arimidex™ Randomized Group Efficacy and Tolerability) study. *J Clin Oncol* **18**: 3748–3757

Boyd S (1900) On oophorectomy in cancer of the breast. *Br Med J* **ii**: 1161–1167

Brodie A, Lu Q, Liu Y and Long B (1999) Aromatase inhibitors and their antitumor effects in model systems. *Endocr Relat Cancer* **6** (2): 205–210

Brodie AMH and Njar VCO (1998) Aromatase inhibitors in advanced breast cancer: mechanism of action and clinical implications. *J Steroid Biochem Mol Biol* **66**: 1–10

Buzdar A, Jonat W, Howell A, Jones SE, Blomqvist C, Vogel CL, Eiermann W, Wolter JM, Azab M, Webster A and Plourde PV for the Arimidex Study Group (1996) Anastrozole, a potent and selective aromatase inhibitor, versus megestrol acetate in postmenopausal women with advanced breast cancer: result of overview analysis of two phase III trials. *J Clin Oncol* **14**: 2000–2011

Buzdar AU, Jonat W, Howell A, Jones SE, Blomqvist C, Vogel CL, Eiermann W, Wolter JM, Steinberg M, Webster A and Lee D for the Arimidex Study Group (1998) Anastrozole versus megestrol acetate in the treatment of postmenopausal women with advanced breast carcinoma: Results of a survival update based on a combined analysis of data from two mature phase III trials. *Cancer* **83**(8): 1142–1152

De-vos D, Mould G and Stevenson D (1989) The bioavailability of tamoxifen: new findings and their clinical implications. *Curr Ther Res* **46**: 703–708

Dowsett M, Goss PE, Powles TJ, Hutchinson G, Brodie AMH, Jeffconte SL and Coombes RC (1987) Use of the aromatase inhibitor 4-hydroxyandrostenedione in postmenopausal breast cancer: optimization of therapeutic dose and route. *Cancer Res* **47**: 1957–1961

Dowsett M, Tobias JS, Howell A, Blackman GM, Welch H, King N, Ponzone R, von Euler M and Baum M (1999a) The effect of anastrozole on the pharmacokinetics of tamoxifen in postmenopausal women with early breast cancer. *Br J Cancer* **79**(2): 311–315

Dowsett M, Pfister C, Johnston SR, Mules DW, Houston SJ, Verbeek JA, Gundacker H, Sioufi A and Smith IE (1999b) Impact of tamoxifen on the pharmacokinetics and endocrine effects of the aromatase inhibitor letrozole in postmenopausal women with breast cancer. *Clin Cancer Res* **9**(9): 2338–2343

Dukes M (1997) The relevance of preclinical models to the treatment of postmenopausal breast cancer. *Oncology* **54** (Suppl 2): 6–10

Early Breast Cancer Trialists' Collaborative Group (1992) Systemic treatment of early breast cancer by hormonal, cytotoxic, or immune therapy. 133 randomised trials involving 31,000 recurrences and 24,000 deaths among 75,000 women. *Lancet* **339**: 1–15, 71–85

Early Breast Cancer Trialists' Collaborative Group (1998) Tamoxifen for early breast cancer: an overview of the randomised trials. *Lancet* **351**: 1451–1467

Geisler J, King N, Dowsett M, Ottestad L, Lundgren S, Walton P, Kormeset PO and Lønning PE (1996) Influence of anastrozole (Arimidex), a selective, non-steroidal aromatase inhibitor, on *in vivo* aromatisation and plasma oestrogen levels in postmenopausal women with breast cancer. *Br J Cancer* **74**: 1286–1291

Geisler J, Bernsten H, Ottestad L, Lindtjorn B, Dowsett M, Lønning PE (1999) Neoadjuvant treatment with anastrozole (Arimidex) causes profound suppression of intra-tumor estrogen levels. *Proc Am Soc Clin Oncol* **18**: 82a Abs 311

Grodin JM, Siiteri PK and MacDonald PC (1973) Source of estrogen production in postmenopausal women. *J Clin Endocrinol Metab* **36**: 207–214

Houghton J and Baum M, ATAC Study Group (1998) 'Arimidex', tamoxifen alone or in combination (ATAC) adjuvant trial in post-menopausal breast cancer. *Eur J Cancer* **34** (Suppl 5): S83 Abs 385. Abstracts from the 1st European Breast Cancer Conference, Florence, 29 Sep-3 Oct 1998

Howell A and Dowsett M (1997) Recent advances in endocrine therapy of breast cancer. *Br Med J* **315** (7112): 863–866

Jaiyesimi IA, Buzdar AU, Decker DA and Hortobagyi G (1995) Use of tamoxifen for breast cancer: twenty-eight years later. *J Clin Oncol* **13**(2): 513–529

Johnston SRD, Haynes BP, Sacks NPM, McKinna JA, Griggs LJ, Jarman M, Baum M, Smith IE and Dowsett M (1993) Effect of oestrogen receptor status and time

© 2001 Cancer Research Campaign 

on the intratumoural accumulation of tamoxifen and N-desmethyltamoxifen following short-term therapy in human breast cancer. *Breast Cancer Res Treat* **28**: 241–250

Jordan VC (1976) Effect of tamoxifen (ICI 46474) on initiation and growth of DMBA-induced rat mammary carcinoma. *Eur J Cancer* **12**: 419–424

Knazek RA, Lippmann ME and Chopra HC (1977) Formation of solid human mammary carcinoma *in vitro. J Natl Cancer Inst* **58**: 419–422

Lien EA, Anker G, Lönning PE, Solheim E and Veland PM (1990) Decreased serum concentrations of tamoxifen and its metabolites induced by aminoglutethimide. *Cancer Res* **50**: 5851–5857

MacDonald PC, Edman CD, Hempsell DL, Porter JC and Siiteri PK (1978) Effect of obesity on conversion of plasma androstenedione to oestrone in postmenopausal women with and without endometrial cancer. *Am J Obstet Gynecol* **130**: 448–455

Miller WR and O'Neill J (1987) The importance of local synthesis of estrogen within the breast. *Steroids* **50**(4–6): 537–548

Mould GP, Stevenson D, Briggs RJ and De-vos D (1986a) A re-evaluation of the pharmacokinetics of tamoxifen. *Acta Pharmacol Toxicol* **59** (Suppl 5): 310 Abs

Mould G, Stevenson D, Briggs RJ, Guelen PJM, Slee PHTJ and De-vos D (1986b) New insights into the tamoxifen pharmacokinetics constitute a basis for a loading dose scheme. *Eur J Cancer and Clin Oncol* **22**: 732 Abs

Nabholtz JM, Buzdar A, Pollak M, Harwin W, Burton G, Mangalik A, Steinberg M, Webster A and von Euler M (2000) Anastrozole is superior to tamoxifen as first-line therapy for advanced breast cancer in postmenopausal women: results of a North American multicenter randomized trial. *J Clin Oncol* **18**: 3758–3776

Yates RA, Dowsett M, Fisher GV, Selen A and Wyld PJ (1996) Arimidex (ZD1033): a selective, potent inhibitor of aromatase in postmenopausal female volunteers. *Br J Cancer* **73**(4): 543–548

 *© 2001 Cancer Research Campaign*
